# Crying spells triggered by thumb-index rubbing after thalamic stroke: a case report

**DOI:** 10.1186/s13104-017-2425-z

**Published:** 2017-02-24

**Authors:** R. Bassani, C. Rosazza, L. Ghirardin, V. Caldiera, E. Banco, C. Casati, L. Tesio

**Affiliations:** 1 Department of Neurological Sciences, Ospedale “G. Salvini”, Garbagnate Milanese, Italy; 2 Department of Neurorehabilitation Sciences, Istituto Auxologico Italiano, IRCCS, Milan, Italy; 3 Department of Neuroradiology, Istituto Neurologico “C. Besta”, IRCCS, Milan, Italy; 4 Department of Biomedical Sciences for Health, Chair of Physical and Rehabilitation Medicine, Università degli Studi di Milano, Milan, Italy

**Keywords:** Crying, Cerebrovascular disease, Thalamus, Stroke, Case report

## Abstract

**Background:**

Pathologic crying, devoid of any emotional counterpart, is known to occur as a consequence of various brain stem, cortical hemispheric and cerebellar lesions or, quite exceptionally, of “dacrystic” epilepsy. The case reported here suggests that thalamic lesions may also cause crying spells, under the special circumstances described below.

**Case presentation:**

After a mild left thalamic stroke a caucasian 77 years old man presented with crying spells with no emotional counterpart, triggered by thumb-index rubbing of his right hand. Only a modest sensation loss on right infra-orbital and nose-labial areas and the first three right fingers could be detected at clinical examination. The circumstances and processes leading to the crying spells were investigated, together with their neural substrate. Brain computerized tomography (CT), magnetic resonance imaging (MRI) and functional magnetic resonance imaging (fMRI) were conducted. Neurophysiologic studies included Video-Electroencephalography, Electromyography, motor and sensory Evoked potentials. Active thumb-index rubbing, passive fingertips stimulation and interaction of sensory-motor stimulation with cognitive/speech activities were tested under different paradigms. A treatment with pregabalin (75 mg twice a day) was attempted. CT and MRI showed a small ischemic infarct in the left ventral postero-lateral thalamus, while fMRI led to the expected findings, i.e. a bilateral activation of the hand motor representation during the crying-triggering right-hand finger rubbing activity. Sensory potentials evoked from stimulation of the right upper limb were the only abnormal neurophysiologic test. Crying spells could be invariably evoked by both real and imagined active finger rubbing, in either the left of right hemi-space. Rubbing by an examiner was ineffective. Immersion in water (18 °C) but not oiling of the fingertips prevented the symptom. Administration and discontinuation of pregabalin 75 mg daily could be associated with suppression and reappearance of the symptom, respectively.

**Conclusions:**

In this patient loss of sensation seemed to generate crying spells rather than the more common allodynia. As a matter of speculation, both symptoms might represent responses to a sensory loss, but in this case the pathway might have been selectively affected providing inhibition from the lateral to the medial segment of the VPLT, which is linked to the anterior cingulate (limbic) cortex engaged in emotional behaviour.

**Electronic supplementary material:**

The online version of this article (doi:10.1186/s13104-017-2425-z) contains supplementary material, which is available to authorized users.

## Background

Despite being rare, pathologic crying, devoid of any emotional counterpart, is known to occur as a consequence of various brain stem and hemispheric cortical lesions [1] or, quite exceptionally, of “dacrystic” epilepsy [2]. To the Authors’ knowledge, lesions of the thalamus has been never suggested as a source of this symptom. The case is presented here of a man suffering from disabling crying spells after a mild thalamic stroke. The symptom was only triggered by thumb-index rubbing of the contralesional hand. This case can stimulate hypotheses concerning the role of the thalamus in crying control.

## Case presentation

A caucasian man aged 77 was admitted to the emergency room with symptoms indicative of a right brachial monoparesis and facio-brachial tingling paresthesiae. Brain computerized tomography (CT) was negative for acute lesions. Approximately 3 h following the onset of symptoms the patient was transferred to a stroke unit where he underwent intravenous thrombolysis with alteplase 0.9 mg kg^−1^.

The procedure was well tolerated with no side effects. The patient was then discharged, despite still suffering of right facio-brachial paresthesiae. A brain CT performed 4 days after the event showed an ischemic lesion to the left ventral-posterior lateral thalamus (VPLT).

**Fig. 1:**
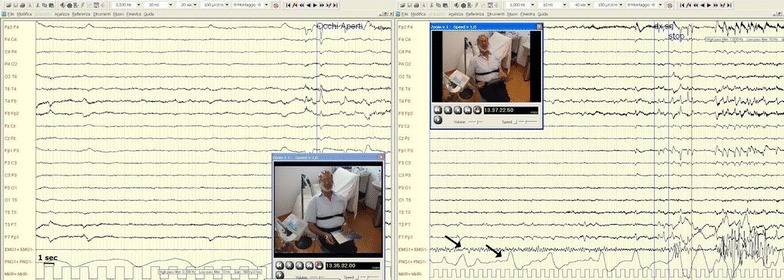
Video-encehalography before (**a**) and during (**b**) thumb-index rubbing of the right hand, causing a crying spell. As soon as the patient starts crying (*left arrow* pointing to the electroencephalographic tracing) the ventilation pattern shows a gradual increment both in frequency and amplitude (*right arrow* pointing to the respiratory tracing)

Two weeks after the event a new symptom was observed by the patient and reported to the first Author. A crying spell, inclusive of tears but devoid of any emotional substrate, was triggered whenever the patient rubbed the tips of his right hand thumb and index fingers. The examiner established through a stopwatch that the frequency of the movement had to be at least 1 Hz. The crying spell started in 5–10 s and faded 3–5 s after the movement stopped. The symptoms were also triggered by various daily activities such as using cutlery and playing cards, thus impairing the patient’s social interaction. No pain was reported. The patient adapted to his condition by wearing a thick glove on his right hand. A series of tests was started 8 weeks after the acute event. Written informed consent was obtained from the patient. Representative motor tests are shown in the video provided with the Additional file 1. Clinical examination showed a moderate sensory loss on light touch on the palmar side of the first three right hand fingers, as well as on the right infra-orbital and nose-labial areas. A video-EEG excluded epileptic seizures during crying spells (Fig. 1). Motor evoked potentials were normal. Sensory evoked potentials from stimulation of the left limbs evidenced increased latency, low voltage and temporal dispersion (not shown).

The patient was administered a neuropsychological evaluation in order to describe his cognitive functioning and to exclude cognitive deficits. Thalamic lesions may in fact lead to many cognitive impairments, spanning from decreased consciousness to dementia and including deficits in spatial representation and memory [3], the latter being of potential relevance for the motor tests described below. However, the cognitive tests ruled out language, praxic, executive, attention, spatial attention, memory and abstract reasoning deficits, as in all tests the patient’s performance was within the tolerance limits of a reference sample of subjects similar for age and educational level (details are provided in the Additional file 1). This was also consistent with the site and the size of the lesion (see below). Active thumb-index rubbing, passive fingertips stimulation and interaction of sensory-motor stimulation with cognitive/speech activities were tested under different paradigms, in blocks as shown in Table 1.

**Table 1:** Motor-sensory tasks aimed at investigating the modality of triggering crying spells under finger scrubbing

Main tasks	Blocks	Task alternative modes	Crying-triggering tasks/modes	On-line video
Light thumb-index rubbing	Eyes open	Left hand; right hand; both	Right hand; both	*
	Eyes closed	Left hand; right hand; both	Right hand; both	
	Eyes closed, imagined movement	Left hand; right hand	Right hand	*
	Eyes open. The “rubbing” hand is covered and replaced by another one’s hand rubbing	Left hand; right hand	Right hand	
	Index and thumb flex but do not touch each other (finger tapping)	Left hand; right hand; both	None	*
	Arms straight and crossed, eyes open	Left hand; right hand; both	Right hand	
	Hand immersion in water, 24 °C	No motion; right hand	None	
	Hand fingertips stained with grease	Right hand	Right hand	
Passive stimulation	Passive rubbing of index fingertip (toothbrush)	Right hand; right hallux	No	
	Warming of hand fingertips (hair dryer)	Right hand	No	
Cognition/speech (narration; reading aloud; month sequencing; counting backwards)		Hands still; right hand “counting money”	Right hand	

* Indicate the tests presented in the Additional file 2

One examiner (CC) stimulated the patient for about 20 s in each task. Another examiner (LT) determined the onset of a crying spell when an evident change in size and/or frequency of ventilation and a typical grimace developed (usually, within 5–6 s). In this case, the stimulation was suddenly stopped. Blocks of tasks were balanced (ABBA sequence). The patient was videotaped. Representative tasks (marked with asterisk in Table 1) are shown in the Additional file 2.

During the experimental session, three independent observers (TL, BR, BE) checked for presence/absence of the crying spell. In Table 1 the fourth column from the right gives the observed outcome (onset/absence of crying spells). This was fully reproducible with no exceptions across repetitions of identical tasks and across observers. It must be highlighted that crying (a) was only triggered by active rubbing of the fingers (not toes), with eyes either open or closed, during either a real or imagined task; (b) it persisted when the fingertips were oiled (olive oil at room temperature 24 °C), but ceased if the hand was immersed in tap water at 18 °C; (c) it appeared irrespective of the fact that the hand was placed in the left or the right hemi-space.

**Fig. 2:**
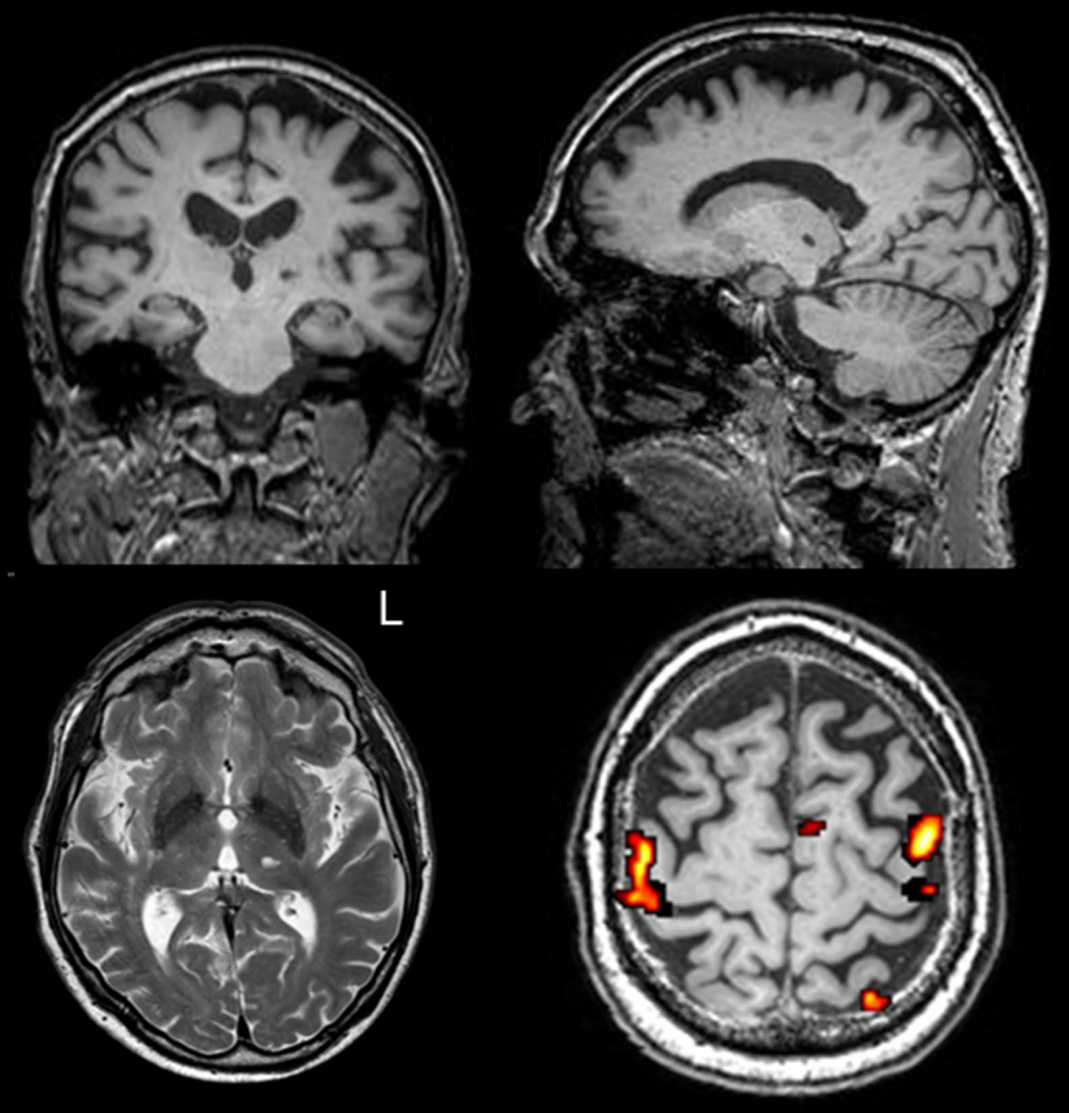
T1 (*upper row* *of panels*) AND T2-weighted (*lower left panel*) magnetic resonance images evidencing a lacunar infarct in the ventral posterolateral portion of the left thalamus. The lesion is millimetric (6 mm longest diameter, 83 mm^3^ volume) and it appears characteristically hypointense in the T1-weighted sequence, and hyperintense in the T2-weighted sequence and in Flair (not shown). Diffusion-weighted MR image and apparent diffusion coefficient map (not shown) do not evidence any associated restriction of water molecules diffusivity, thus confirming that the lesion is a chronic lacunar stroke. In addition, the magnetic resonance imaging shows the presence of multiple gliotic subcortical and periventricular areas suggestive of a chronic microvascular ischemic disease, a common finding in elderly people. In the *lower right corner the figure* shows functional magnetic resonance images of a sensory-motor task consisting of rubbing the fingers of the affected (*right*) hand. The movement elicited a typical pattern of activation of both the controlateral motor and sensory hand area and the ipsilateral hand area (P < 0.001, uncorrected). Images are all shown in radiological convention (*right* is *left*)

The MRI exam showed an old lacunar infarct in the left thalamus, likely involving its ventral, posterolateral portion (VPLT, Fig. 2). This small lesion (6 mm largest diameter, 83 mm^3^ volume) appeared characteristically hypointense in the T1-weighted image (lower left image) and hyperintense in the T2-weighted image (lower right image). MRI also showed signs of chronic microvascular ischemic disease, a common finding in elderly people.

An fMRI exam was also performed (Fig. 2). A blocked-design sensory-motor task was adopted, with 12 active blocks (6 for each side) alternating with 8 rest blocks. The task consisted of active thumb-index rubbing on visual instruction, for 20 s per block. Two examiners (CR, LT) checked the patient’s performance. The patient was instructed to stop rubbing when crying started (usually within 5–6 s) in order to avoid head movements hindering the results of the fMRI task. The sensory-motor task elicited a typical pattern of activations in the motor and sensory hand area, which were weaker for the affected (right) hand (P < 0.001, uncorrected) as the movement was only briefly performed. Presumably, this asymmetry also reflected the longer stimulation allowed to the unimpaired hand. During movement of the impaired hand, the pattern of activation observed with fMRI involved expected areas, i.e. both the controlateral motor and sensory hand area and the ipsilateral hand area. Pregabalin was prescribed (75 mg the first day, then 75 mg twice daily). The crying spells receded within a week. The drug, however, caused some sedation. After the first week it was gradually suspended through the subsequent week. Crying spells reappeared the day after the treatment was discontinued. The drug was re-administered and crying spells disappeared again in one week. Under continuous treatment no crying spells were reported during a 6 months period.

## Conclusions

An ischemic lesion of the VPLT was the only one visible brain alteration that could be related to patient’s symptoms. Presumably, this resulted from occlusion of one or more of the thalamogeniculate arteries [4]. The fMRI findings were all expected.

The sensory deficit closely matched the one seen in cases of hand-mouth (“cheiro-oral”) sensory syndromes caused by a lesion in the VPLT, where representations of the face and of the hand are very close to each other [5]. How to classify this case, within the framework of known neurologic syndromes? The choice depends on whether the sensory or the behavioural side of the phenomenon are highlighted. On the sensory side, two hypotheses can be put forward: the case is an example of (a) thalamic (Roussy-Dejérine) syndrome [6], or (b) of allodynia within a poststroke thalamic pain (PSTP) syndrome [7].

- Classifying the case within the “analgetic” subtype of thalamic syndromes fits with the lesion site, the absence of pain, and the moderate alterations in sensation and sensory evoked potentials.
- A subclinical mechanical allodynia [8] was seemingly at work. The fingertips stimulation seemed to elicite much more than a pure feeling of touch. While allodynia is a component of the PSTP syndrome, yet it belongs to the large family of neuropathic pain symptoms and is not necessarily associated with thalamic lesions. It may follow pure sensory loss and it is considered to reflect central “sensitization” stemming from a slow maladaptive plasticity [7, 9]. As it is the case for most of the allodynic symptoms, also in this case crying appeared weeks after the lesion. Consistently with this sensory-allodynic interpretation, suppression of the symptom could be obtained by immersing the hand in water at 18 °C for a few seconds. This possibly followed a cooling-related mild increase of the threshold for touch detection [10], and indeed lubrication with oil was unsurprisingly ineffective. Presumably, the mere lubrication (at 24 °C) did not cool the skin and left the tactile threshold unaffected, although in itself it can influence grip forces and the sense of touch pleasantness [11]. In addition, the symptom was highly responsive to pregabalin, known to be effective in neuropathic pain [12].

Neither hypothesis fits perfectly the clinical picture, however, due to the absence of pain.

If the crying behaviour per se is highlighted, three more candidate classifications for this case can be put forward. The case could represent a variant of (a) “disorder of laughing and crying”—DLC [13], (b) of release of the palmo-mental reflex and (c) of tickle behaviour.

- The case might be reasonably classified as a “negative sensory” variant of DLC. In fact the underlying pathology is of destructive, not irritative (e.g. epileptic), nature (hence the “negative” connotation) and the symptom is triggered by stimuli usually unrelated to crying. The mechanism leading to this type of DLC is described by its proponents as follows: “Destructive lesions or diaschisis alter central appraisal and sensory processing of feelings or emotional or nonemotional stimuli, leading to inappropriate emotional output after a stimulus, so that stimuli such as pain and touch lead to inappropriate emotional output” [13]. The new classification is consistent with the observation that a variety of diseases and lesion sites, possibly affecting shared neural circuits, may lead to similar clinical pictures. In fact, the proponents state that “the concept of laughter and crying *centers* should be replaced by *circuits* modulating emotional and sensory stimuli” (italics in the original text) [13].

Neither this classification can encase the whole clinical picture, however. The fact that crying was only triggered by active (self) thumb-index rubbing, both physical and imagined, remains in fact unexplained.

b. Rubbing of the thenar eminence may be sufficient to trigger the palmomental reflex [14], both in healthy infants and in adults after various brain lesions.

Again, the above classification does not fit the clinical picture. In fact, the palmo-mental reflex can be elicited by passive (e.g. by another person) rubbing.

c. In tickle, mild cutaneous stimuli (including rubbing of the palms) may evoke contradictory feelings (both pleasant and unpleasant) and complex avoidance behaviours. It is known that self-tickling is impossible [15]. By contrast, crying spells in the case reported here could be only evoked by active self-rubbing.

Two main questions thus remain open: (a) why finger rubbing elicited crying and neither laughing nor pain and (b) why only self-rubbing, either real or imagined, and not passive rubbing could trigger the crying spells.

With respect to the former question, one should recall that laughing and crying both belong to the DLC syndromes. A sudden shift from crying to laughing can be oserved in the same patient, in response to the same stimulus (for historical recalls on pathologic laughter and crying see ref. 1). The site of the crying/laughing switch (or switches) is presently unknown. For sure, crying and pain are related. For unknown reasons crying is perhaps the most primitive and explicit behaviour communicating pain and other danger-related feelings such as hunger and fear [16]. Yet, in many circumstances healthy adults can suppress crying. Actually, the behavioural and neurobiological basis of inappropriate, “pathologic” crying still remains poorly understood [17].

This curious case encourages some speculation on the site of the damage to the neural circuitries linking (or, possibly, decoupling) cutaneous sensation and crying. The lateral thalamus projects to the primary and secondary somatosensory cortices and is considered to be involved in the discriminative and thermal-nociceptive dimensions of pain. The medial thalamus (so called “limbic” thalamus) [18] projects to the anterior cingulate cortex and is considered to control, through this projection, the affective-emotional and behavioural aspects of pain. In fact, various models of deafferentation pain, typically presenting with severe emotional characteristics, converge in ascribing symptoms to hypoactivity of the lateral thalamus, becoming less effective in inhibiting the medial thalamus and therefore, indirectly, the anterior cingulate cortex [7]. In fact in mammals the cingulate gyrus, also said the limbic cortex, is part of the crying circuitry which, in its purely motor component, in all vertebrates is essentially a brain stem circuitry. The anterior cingulate cortex is hyperactive in mechanic hyperalgesia in complex regional pain syndromes [19], which are exacerbated by incongruent sensory motor actions as previously highlighted. The thalamo-cingulate pathway is one of the three major divisions of the limbic system (the other being the amigdalar and the septal branches). In the patient described in this work, the thalamic lesion evidenced through MRI is located in the VPLT, presumably in the small area where both the face and the hand are represented. As a matter of speculation, the lesion might have selectively affected the inhibitory lateral-to-medial thalamus projection, thus ultimately dishinibiting the cingulate cortex and, indirectly, the crying behaviour. By contrast, the projection from the lateral thalamus to the primary and secondary cortices might have been relatively spared. This would explain why pregabalin, a drug which decreases both neuronal hyperexcitability and neuropathic pain, was effective also in preventing crying.

In 1923 it was proposed that “dishinibtion” of the brain stem circuits subtending crying could arise from lesion of areas of the motor cortex specifically dedicated to the involuntary (emotional) control of brain stem circuits [20]. This hypothesis has been recently challenged in favour of a cerebellar origin of the symptom [1] following a direct lesion or a lesion of afferents originating from the prefrontal/anterior cingulate cortex. This should imply, however, a deficit in spatio-motor problem solving tasks, such as the Stroop and the Tower of Hanoi tests [1]. The Stroop and the simplified version of the TOH (the London Tower test), however, were normal in this case (see table in Additional file 1). Also, both the MRI and the fMRI exams did not provide evidence of alterations in thalamo-frontal nor thalamo-cerebellar pathways.

With respect to the second question (asking why active, not passive, rubbing was effective) one can again speculate that the sensory deficits created a mismatch between the expected and the actual sensory feedback arising from movement. The so-called action-effect binding [21] is the basis for the “sense of agency”, the perception that sensation was caused by one’s own action. A frustrated sense of agency, both in real and imagined movements, is a potential source of pain in complex regional pain syndromes [22, 23]. Sensory-motor incongruence can also exacerbate chronic pain after whiplash injury [24]. On the other hand, re-matching delusive movements and providing the expected visual feedback from an amputated limb can decrease phantom limb pain [25, 26].

In a study with Positron Emission Tomography, other cortical regions have been proven to be involved in perceiving a sensory mismatch (e.g. between vision and proprioception) and in controlling a movement under sensory-motor mismatch. These are the right ventral lateral prefrontal cortex and the right dorsal lateral prefrontal cortex, respectively [27]. Again, in the present case no alterations were detected in these areas at MRI examination, and at fMRI examination during the thumb-index rubbing task. Only the Supplementary Motor Area of Area 6 was activated, as a typical pattern elicited during a simple hand movement. Presumably, premotor or parietal activation would have been be observed if visual information was presented [27]. Of course, anatomical lesions and circuital impairments undetectable through MRI and/or fMRI could not be ruled out.

To sum up, a disinhibition of the thalamo-limbic pathways remains a plausible-although, unproven-explanation for the phenomenon. In this case the lesion might have been moderate enough to make the limbic activation undetectable at fMRI and the crying spell to require the simultaneous double triggering of the limbic areas, i.e. from peripheral stimuli causing hyperexcitation of the (already dishinibited) medial thalamic circuits, like in allodynia, and from direct central hyperexcitation of the limbic cortex caused by a frustrated sense of agency, like in other pain syndromes.

## Additional files

**Additional file 1.** Neuropsychological assessment. Table S1 contains the list of tests of neuropsychological assessment, including: (a) Test names. (b) Scores. (c) References to each of the listed tests.

**Additional file 2.** Crying spells triggered by thumb-index rubbing after thalamic stroke. It contains the video of the patient during “index-thumb rubbing” manoeuvres, showing his crying spells.

## Abbreviations

CT: computerized tomography
EEG: electroencephalografy
EMG: electromyography
fMRI: (functional) magnetic resonance imaging
MRI: magnetic resonance imaging
VPLT: ventral postero-lateral thalamus
