# Supplementary material for: Crying spells triggered by thumb-index rubbing after thalamic stroke: a case report
Source: BMC Res Notes. 2017 Feb 24;10:109. doi: 10.1186/s13104-017-2425-z (PMC5326498; doi:10.1186/s13104-017-2425-z)
Supplement: Supplementary file 1 — Additional file 1. Neuropsychological assessment. Table S1 contains the list of tests of neuropsychological assessment, including: (a) Test names. (b) Scores. (c) References to each of the listed tests. [file 13104_2017_2425_MOESM1_ESM.docx]

**Neuropsychological assessment**

The patient underwent neuropsychological assessment (Table S1). He was alert, cooperative and oriented in space and time (a). No aphasic deficits were found. Tests on language (b,c,d,e,f), memory span (g,h), executive and attentive functions (i,j,k), praxia (l,m,n,o) and reasoning (p) were all in the standard.

TABLE S1

|  | **range** | **raw score** | **age/education -corrected score** | **cutoff** | **equivalent score** |
| --- | --- | --- | --- | --- | --- |
|  |  |  |  |  |  |
| a. Mini-Mental State Examination MMSE [1] | 0-30 | 27 | 26 | 22 |  |
| *Language* |  |  |  |  |  |
| b. Token Test [2] [3] | 0-36 | 32 | 31.5 | 26.5 | 2 |
| c. E.N.P.A. battery [4] |  |  |  |  |  |
| - reading |  |  |  |  |  |
| -word | 0-10 |  |  | 6.4 |  |
| - no-word | 0-5 |  |  | 4.0 |  |
| - sentences | 0-2 | 2 | 2 | 1.3 |  |
| d. Phonemic fluency [3] | 0-∞ | 26 | 26 | 17 | 2 |
| e. Semantic fluency [3] | 0-∞ | 44 | 46 | 25 | 4 |
| f. Boston Naming Test [5] | 0-60 | 50 |  | 43 |  |
| *Memory* |  |  |  |  |  |
| g. Digit Span [6] | 0-9 | 6 | 6 | 3.75 | 4 |
| h. Corsi’s visuo-spatial span [6] | 0-9 | 6 | 6.25 | 3.5 | 4 |
| *Executive and attentional functions* |  |  |  |  |  |
| i. Selective attention, barrage test [2] | 0-60 | 59 | 59.5 | 31 | 4 |
| j. Stroop’s Test [7] |  |  |  |  |  |
| - time interference | 0-∞ | 30 | 17.5 | ≤36.91 | 4 |
| - error interference | 0-30 | 0.5 | 0 | ≤4.23 | 4 |
| k. Simplified London Tower Test [8] | 0-18.80 | 18.8 | 18.8 | 9.25 | 4 |
| *Praxia* |  |  |  |  |  |
| l. Ideomotor praxia [9][10][11] | 0-72 | 63 |  | 53 |  |
| - symbolic gesture | 0-30 | 26 |  |  |  |
| - non-symbolic gesture | 0-42 | 37 |  |  |  |
| - static gesture | 0-36 | 36 |  |  |  |
| - dynamic gesture | 0-36 | 27 |  |  |  |
| m. Ideational praxia [9][10][11] |  |  |  |  |  |
| - use of objects | 0-5 | 5 |  | - - |  |
| n. Orofacial praxia [9][10][11] | 0-24 | 21 |  | 20 |  |
| o. Constructive praxia [2] [9][10][11] | 0-14 | 12 | 12 | 8 | 3 |
| *Reasoning* |  |  |  |  |  |
| p. Raven coloured progressive matrices [12] | 0-36 | 29 | 33.5 | 18 | 4 |

REFERENCES

1 Grigoletto F, Zappalà G, Anderson D, Lebowitz B. Norms for the Mini-Mental State Examination in a healthy population. Neurology 1999; 53: 315-20.

2 Spinnler H, Tognoni G. Standardizzazione e taratura italiana di test neuropsicologici. Ital J Neurol Sci 1987; (Suppl. 8).

4 Capasso R, Miceli G. Esame neuropsicologico per l’afasia (E.N.P.A.). In: Caltagirone C, Razzano C, editors. Metodologie riabilitative in logopedia. Milano: Springer-Verlag; 2001.

3 Novelli G, Papagno C, Capitani E, Laiacona M, Vallar G, Cappa SF. Tre test clinici di ricerca e produzione lessicale. Taratura su soggetti normali. Arch Psicol Neurol Psich 1986; 47,4: 477-506.

5 Kaplan E, Goodglass H, Weintraub S. Boston naming test. Philadelphia: Lea & Febiger; 1983.

6 Orsini A, Grossi D, Capitani E, Laiacona M, Papagno C, Vallar G. Verbal and spatial immediate memory span: Normative data from 1355 adults and 1112 children. Ital J Neurol Sci. 1987; 8,6: 537-48.

7 Caffarra P, Vezzadini G, Dieci F, Zonato F, Venneri A. Una versione abbreviata del test di Stroop: Dati normativi nella popolazione italiana. Riv Neurol. 2002; 12: 111-5

8 Allamano N, Della Sala S, Laiacona M., Passetti C, Spinnler H. Problem solving ability in aging and dementia: normative data on a non-verbal test. Ital J Neurol Sci. 1987 8:111-20.

9 De Renzi E, Motti F, Nichelli P. Imitating gestures: A quantitative approach to ideomotor apraxia. Arch Neurol. 1980; 37,1: 6-10.

10 De Renzi E, Lucchelli F. Ideational apraxia. Brain 1988; 111: 1173-85.

11 De Renzi E, Faglioni P. L’aprassia. In: Denes G, Pizzamiglio L, editors. Manuale di neuropsicologia. 2nd edition Bologna: Zanichelli; 1996. p.557-93.

12 Basso A, Capitani E, Laiacona M. Raven’s coloured progressive matrices: normative values on 305 adult normal controls. Funct Neurol 1997; 2, 2:189- 94
